# Radiation-induced intestinal injury: from molecular mechanisms to clinical translation

**DOI:** 10.3389/or.2025.1613704

**Published:** 2025-08-29

**Authors:** Wenjue Wu, Yubo Cai, Zhi Yang, Mengshuang Chen, JianYang Hu, Kunlong Qu, Jian Yang

**Affiliations:** Zhejiang Cancer Hospital, Hangzhou Institute of Medicine (HIM), Chinese Academy of Sciences, Hangzhou, Zhejiang, China

**Keywords:** radiation-induced intestinal injury, pathogenesis, diagnosis, biomarkers, treatment strategies

## Abstract

Radiation-induced intestinal injury (RIII) poses a significant clinical challenge for patients undergoing pelvic or abdominal radiotherapy, characterized by dual features of acute symptoms (diarrhea, abdominal pain, rectal bleeding) and chronic complications (stricture, fistula, chronic pain), profoundly impacting quality of life. Despite high clinical prevalence, the molecular and cellular mechanisms underlying RIII remain poorly defined, hindering therapeutic development. Current diagnostic modalities (imaging, endoscopy) lack sensitivity and specificity for early detection or real-time monitoring. While biomarkers offer promise for non-invasive assessment and prognosis, existing candidates face limitations in reproducibility and clinical applicability. Therapeutic options, ranging from pharmaceuticals to surgery, show variable efficacy, underscoring the need for optimized strategies. This review systematically explores RIII pathogenesis, emphasizing radiation-induced immune dysregulation, epigenetic alterations, and gut microbiota dysbiosis. We discuss potential biomarkers, such as miRNA, fatty acid binding proteins (FABPs), etc. We categorize therapies into radioprotectors (pre-radiation use) and radiomitigators (post-radiation intervention), highlighting natural plant-derived compounds and traditional Chinese medicine (TCM) for their multi-target effects, alongside emerging approaches like stem cell and microbiota transplantation, with discussions on their therapeutic potential and clinical challenges. Crucially, we exclusively summarize recent clinical translation advances to accelerate drug development. Through critical evaluation of evidence, we propose future directions to refine risk stratification, enable timely intervention, and improve long-term outcomes for irradiated patients. This integrative analysis aims to bridge translational gaps and prioritize research avenues for RIII management.

## 1 Introduction

Radiation therapy stands as one of the most effective modalities for cancer treatment, applicable to over half of all cancer patients (1). However, ionizing radiation (IR) can induce varying degrees of damage to the human body. The small intestine, with its rapid cellular turnover, is highly sensitive to radiation. Gastrointestinal injury typically occurs when the entire intestinal tract is exposed to radiation doses exceeding 5 Gray (Gy) (2). Consequently, clinical radiotherapy targeting abdominal/pelvic tumors frequently leads to radiation-induced intestinal injury (RIII). Currently, over 75% of patients develop acute RIII within hours to weeks post-irradiation, manifesting as symptoms such as bleeding, pain, or diarrhea (3, 4). Furthermore, 5%–20% of patients progress to chronic RIII months or years later, characterized by irreversible tissue damage, intestinal fibrosis, or strictures (3, 4). In severe cases, RIII may result in intestinal necrosis, perforation, or even death (5, 6), underscoring its significant impact on long-term cancer survivors’ quality of life and the urgent need for effective management strategies.

While advanced imaging modalities such as abdominopelvic computed tomography (CT), magnetic resonance enterography (MRE), and confocal laser endomicroscopy (CLE) provide critical insights into morphological changes, their reliance on anatomical visualization restricts early functional assessment and prognostication (7, 8). With the in-depth study of the pathogenesis, more and more potential biomarkers have been discovered, which may become non-invasive assessment and prognosis prediction methods for RIII. The emergence of molecular diagnostics has renewed interest in identifying biomarkers capable of non-invasive detection and risk stratification. Preclinical and early clinical studies have identified promising candidates, including microRNAs, circulating exosomes (EVs), and gut microbiome diversity, which correlate with disease severity and treatment response.

Despite these advances, current management strategies remain predominantly palliative, relying on anti-diarrheal agents, corticosteroids, and surgical interventions that fail to address the underlying pathophysiology of RIII (7). This therapeutic gap highlights the urgent need for precision medicine frameworks that integrate biomarker-driven stratification and mechanism-targeted therapies (6).

**FIGURE 1:**
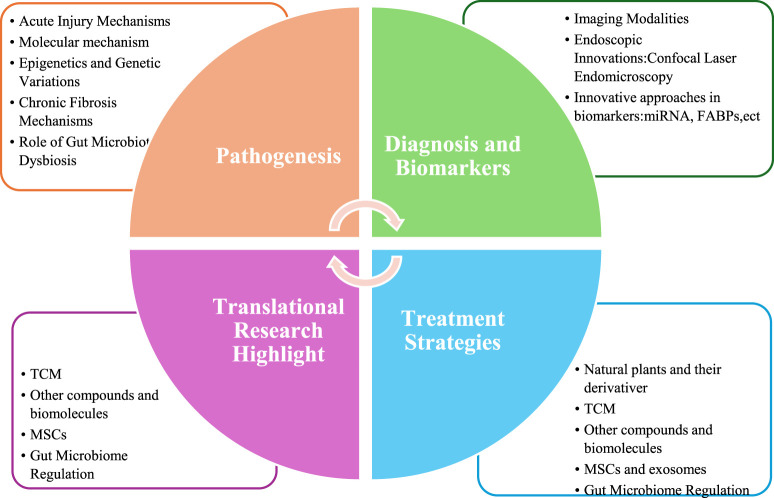
The main content of the document.

Recent preclinical and clinical studies have achieved significant progress, demonstrating that natural plants and their derivatives, traditional Chinese medicine (TCM), drug repurposing, mesenchymal stem cells (MSCs), and gut microbiome regulation can all serve as potential treatments for RIII. Some preclinical studies have laid the groundwork for translating experimental therapies into clinical practice (9–16). In this review, we synthesize the current understanding of RIII pathogenesis, evaluate emerging biomarkers with diagnostic and prognostic potential, and critically assess innovative treatment modalities. By integrating molecular insights with clinical translation, we aim to outline future directions for optimizing RIII management and improving long-term patient outcomes. The main content of the entire document consists of the following four parts (*Pathogenesis*, *Diagnosis and Biomarkers*, *Treatment Strategies* and *Translational Research Highlight*) as shown in Figure 1, and the last part is about the future exhibition booths (not included in Figure 1).

## 2 Pathogenesis

### 2.1 Acute injury mechanisms

**FIGURE 2:**
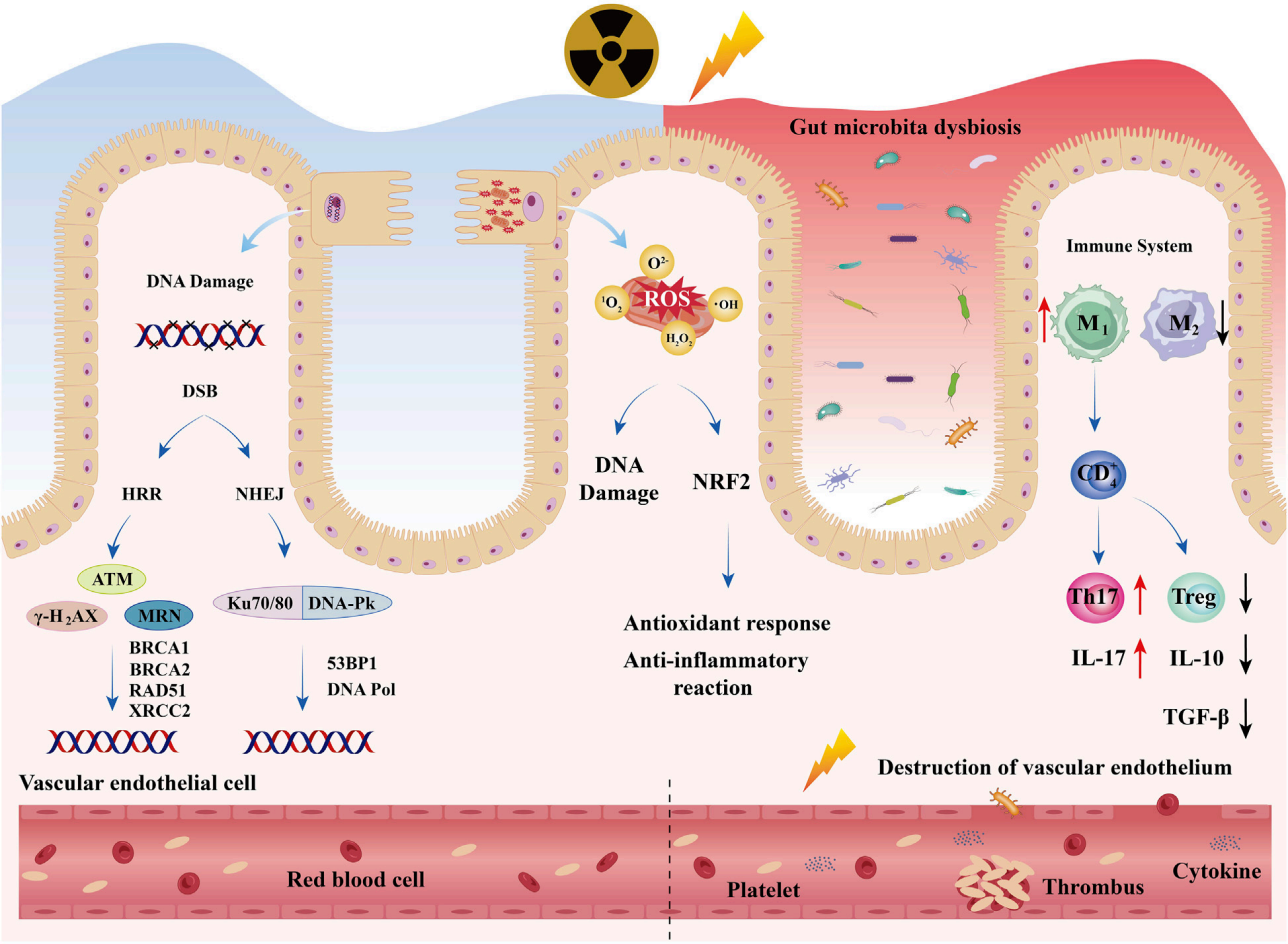
Acute Injury Mechanisms: RIII is a complex process that begins with intestinal cell damage and involves an interplay of direct DNA damage, oxidative stress, and dysregulated immune response. Meanwhile, radiation also causes dysbiosis of the intestinal microbiota and damage to vascular endothelium.

RIII is a complex process that begins with intestinal cell damage and involves an interplay of direct DNA damage, oxidative stress, and dysregulated immune responses (Figure 2).

IR induces the formation of clustered DNA lesions, encompassing double-strand breaks (DSBs), single-strand breaks (SSBs), and base modifications (17). These lesions trigger the activation of the DNA damage response (DDR) pathway, which involves a cascade of molecular events including sensor proteins (MRN [MRE11-RAD50-NBS1] complex), signal transducers (ATM [Ataxia-Telangiectasia Mutated], ATR [ATM and Rad3-related], DNA-PKcs [DNA-dependent protein kinase catalytic subunit]), and downstream effectors (P53, CHK1/2 [Checkpoint kinase 1/2]). In the absence of proper repair mechanisms, these lesions may result in irreversible cellular damage or programmed cell death (18). The molecular mechanisms underlying these processes will be comprehensively elucidated in subsequent sections.

Concurrently, oxidative stress, mediated by reactive oxygen species (ROS), exacerbates DNA damage and disrupts cellular homeostasis (19). Ionizing radiation(IR) induces the radiolysis of water molecules, generating hydroxyl radicals (·OH), superoxide anions (O_2_^−^), and hydrogen peroxide (H_2_O_2_), which subsequently attack cellular components including lipids, proteins, and nucleic acids (20).

Dysfunction of the immune system is also a critical factor in the initiation and progression of RIII (21, 22). The intestinal mucosa and submucosa harbor numerous innate immune cells, including macrophages and natural killer cells (23). Macrophages bridge innate and adaptive immune responses and can undergo polarization in response to IR, differentiating into pro-inflammatory (M1) or anti-inflammatory (M2) phenotypes (24). In murine models, IR promotes the activation of pro-inflammatory M1 macrophages while reducing anti-inflammatory M2 macrophages. Polarized macrophages secrete cytokines that activate T cells and induce adaptive immune responses. Immature cluster of differentiation four positive T lymphocyte (CD4^+^ T) cells can directly differentiate into helper T (Th) cell subsets (Th1, Th2, Th17) and regulatory T cells (Treg) (25).

The dysregulation of the Th17/Treg equilibrium represents a fundamental characteristic of inflammatory diseases (26). This pathological phenomenon has been extensively documented in total abdominal irradiation murine models, where advanced analytical techniques including flow cytometry and immunofluorescence staining have consistently demonstrated that ionizing radiation significantly upregulates intestinal Th17 cell populations while concurrently downregulating Treg cell populations (27). Th17 cells actively secrete pro-inflammatory cytokines, particularly interleukin (IL)-17, which subsequently induces the production of IL-6 and tumor necrosis factor (TNF)-α through positive feedback mechanisms. Conversely, Treg cells are responsible for the secretion of anti-inflammatory cytokines, specifically IL-10 and transforming growth factor (TGF)-β. These molecular events collectively contribute to the breakdown of the mucosal barrier integrity, enhancement of intestinal permeability, and facilitation of bacterial translocation, ultimately resulting in the amplification of inflammatory cascades and exacerbation of tissue damage.

### 2.2 Molecular mechanism

Serval study (28, 29) reveals that most IR-induced genes depend on a limited number of sensors and pathways, especially the DNA damage-activated kinase ATM, DNA-PK, the tumor suppressor P53, transcription factor nuclear factor kappa-light-chain-enhancer of activated B cells (NF-κB) and the ROS-induced transcription factor nuclear factor erythroid 2-related factor 2 (NRF2). Furthermore, X-ray cross-complementing (XRCC) genes represent one of the most extensively investigated categories within the DNA repair gene family. The intricate mechanisms associated will be comprehensively elucidated in the subsequent sections.

Primarily, DSBs are primarily repaired through two distinct mechanisms: homologous recombination repair (HRR) and non-homologous end joining (NHEJ). DNA-PKcs functions as a crucial kinase in the NHEJ pathway, orchestrating both end processing and the assembly of the repair complex (30, 31). The DNA-PK complex is structurally composed of three key components: the catalytic subunit (DNA-PKcs) and two regulatory subunits (Ku70 and Ku80). Upon the occurrence of DSBs within a cell, the Ku70/Ku80 heterodimer exhibits rapid recognition capability and subsequently binds to the damaged DNA termini. This Ku complex then facilitates the recruitment of DNA-PKcs to the site of DNA damage, thereby assembling the complete and functional DNA-PK complex (32). Following its activation, DNA-PKcs initiates the DNA repair cascade through a series of molecular events, including autophosphorylation and the phosphorylation of various downstream protein substrates.

#### 2.2.1 ATM

Recently, an investigation of RIII has revealed that the deubiquitinase USP15 serves as a pivotal regulator of ATM kinase stability. USP15 facilitates the stabilization of ATM through the deubiquitination of K48-linked polyubiquitin chains, thereby augmenting DNA damage repair (33). As we all know, ATM serves as a pivotal kinase in the DDR.

Upon DNA damage, ATM undergoes autophosphorylation and dissociates into active monomeric forms (31, 34, 35). It coordinates the DDR by phosphorylating diverse downstream targets, including:

a. DNA Repair: ATM kinase orchestrates the equilibrium between breast cancer type 1 susceptibility protein (BRCA1)-mediated HRR and TP53-binding protein 1(53BP1)-mediated NHEJ via the ATM/ATM and Rad3-related (ATR)- checkpoint kinase 1(Chk1) signaling axis (36–39).
b. Cell Cycle Regulation: ATM activates checkpoint kinases CHK2 and P53 to induce cell cycle arrest, allowing time for DNA repair. If damage is irreparable, ATM promotes apoptosis to prevent transmission of damaged DNA (40, 41).
c. Transcriptional Regulation: ATM kinase orchestrates the induction of interferon (IFN)-related gene expression through the activation of interferon regulatory factor 1 (IRF1) and concurrently stimulates the NF-κB signaling pathway, thereby modulating inflammatory and immune responses (42, 43). It also suppresses stimulator of interferon genes (STING)-mediated chronic IFN responses to balance immune homeostasis (29).
d. Antioxidant Response: ATM activates transcription factors such as NRF2 to counteract oxidative stress (44).

#### 2.2.2 P53

The P53 protein, encoded by the TP53 gene, is one of the most critical tumor suppressor proteins within cells, renowned as the “Guardian of the Genome.”

DNA damage in intestinal epithelial cells can impede the ubiquitination process of P53, resulting in its activation and subsequent formation of P53 tetramers. These tetramers translocate to the nucleus, where they bind to specific DNA sequences and initiate the transcription of genes associated with apoptosis and cell cycle regulation (29). Notably, the radiation response of intestinal stem/progenitor cells exhibits differential regulation of both apoptosis-dependent and apoptosis-independent mechanisms mediated by P53 (45, 46). Specifically, the cell cycle inhibitory protein P21 plays a crucial role in preventing the proliferation of cells harboring mutated or damaged DNA by inducing G1 phase cell cycle arrest and initiating DNA repair processes (45). In contrast, P53 can also promote apoptosis through the transcriptional activation of pro-apoptotic genes, including P53 upregulated modulator of apoptosis (Puma), bcl-2-associated X protein (Bax), and oxidative stress-activated apoptosis (Oxa) (46).

The normal maintenance or post-injury regeneration of rapidly renewing tissues (including the gastrointestinal tract) relies on specific stem cell subpopulations (47). Studies demonstrate that PUMA gene-deficient mice exhibit greater protection of colon crypt base columnar cells (CBCs) against IR-induced apoptosis compared to P53-deficient mice (48, 49). Enhanced DNA repair and genomic stability mediated by P2 may further promote effective regeneration while preventing P53 activation-induced depletion of adult stem cells (50). Currently, no effective interventions exist against acute radiation-induced gastrointestinal injury. Small-molecule PUMA inhibitors may represent a novel therapeutic approach.

#### 2.2.3 NF-κB

NF-κB, a family of highly conserved transcription factors, serves as a pivotal regulator in diverse biological processes, including immune responses, inflammatory reactions, cell survival, proliferation, and oxidative stress modulation (51). The activation mechanism of NF-κB is dependent on the phosphorylation of the IκB kinase (IKK) complex. Specifically, NF-κB facilitates the transcriptional activation of proinflammatory genes, such as TNF-α, IL-1β, IL-6, and cyclooxygenase (COX)-2, consequently orchestrating the intestinal inflammatory cascade triggered by IR (52).

Numerous investigations have systematically examined the impact of NF-κB inhibition on radiosensitivity across various experimental models (52–55). Particularly noteworthy are the findings from studies utilizing IKKβ-deficient intestinal epithelial cells, which demonstrated enhanced apoptotic activity associated with increased expression and activation of the tumor suppressor protein P53, concomitant with reduced levels of pro-survival Bcl-2 family members. These comprehensive results highlight the essential function of the NF-κB signaling pathway in maintaining intestinal epithelial integrity against radiation-induced cellular apoptosis *in vivo*, thereby establishing IKKβ as a crucial therapeutic target for the development of radioprotective interventions in the gastrointestinal tract (56).

Intriguingly, both ATM and DNA-PK exhibit dual regulatory capabilities, facilitating pro-apoptotic responses through P53 stabilization while simultaneously promoting anti-apoptotic responses via NF-κB activation (57). Furthermore, research has demonstrated that NF-κB can modulate the expression of the pro-apoptotic protein Bax, which is typically induced by P53 in colon cancer cells (58). These two transcription factors, NF-κB and P53, predominantly exhibit antagonistic functions (57), potentially competing for transcriptional co-activator interactions (57, 59). Notably, in certain cellular contexts, NF-κB appears to be essential for mediating the apoptotic effects initiated by P53 (60), highlighting the intricate and context-dependent nature of the interplay between these two signaling pathways.

#### 2.2.4 STING

STING, a crucial intracellular signaling molecule, serves as a pivotal regulator in the innate immune system. IR induces DNA damage, which initiates the recognition of cytosolic DNA by cyclic GMP-AMP synthase (cGAS), thereby activating STING. Following this activation, TANK-binding kinase 1 (TBK1) phosphorylates IRF3, culminating in the expression of IFN-β. This cascade constitutes the cGAS-STING signaling pathway, which contributes to chronic inflammation and tissue damage, thereby exacerbating the pathological progression of RIII (61).

#### 2.2.5 NRF2

NRF2, a pivotal transcription factor within the Cap’n’collar (CNC) family, serves as a master regulator of cellular antioxidant stress responses, detoxification metabolism, mitochondrial biogenesis, and autophagy, thereby playing an indispensable role in radiation protection (62). ROS initiate the dissociation of NRF2 from the Kelch-like ECH-associated protein 1 (KEAP1) complex, resulting in its activation (63). Upon activation, NRF2 orchestrates the transcriptional upregulation of downstream antioxidant genes, including NAD(P)H quinone oxidoreductase 1 (NQO1), superoxide dismutase 2 (SOD2), and glutathione (GSH), which collectively facilitate the repair of oxidatively damaged DNA, lipids, and proteins. Additionally, NRF2 directly modulates gene expression by binding to the antioxidant response element (ARE) within their promoter regions (64, 65).

In NRF2- deficient mice (61), intestinal tissues demonstrated markedly increased levels of cGAS, phosphorylated STING (pSTING), phosphorylated TBK1 (pTBK1), phosphorylated IRF3 (pIRF3), and IFN-β, while NRF2 overexpression downregulated these protein expressions. NRF2 knockdown in human intestinal epithelial cells (HIEC) led to activation of the cGAS-STING pathway and exacerbated DNA damage; in contrast, NRF2 overexpression or Pirin overexpression mitigated these effects. This study demonstrates that NRF2 directly upregulates Pirin gene expression through binding to ARE, consequently inhibiting the cGAS-STING pathway and attenuating RIII.

#### 2.2.6 XRCC

Polymorphisms in XRCC genes may impair their ability to repair radiation-induced DNA damage, thereby contributing to the development of radiation enteropathy. Studies have demonstrated that specific XRCC variants are associated with reduced DNA repair efficiency and increased cellular susceptibility to radiation exposure.

Notably, the XRCC1 399 Arg/Gln polymorphism has been shown to possess significant predictive value for severe acute adverse effects induced by radiotherapy, particularly in cases of acute mucositis and gastrointestinal toxicity (66). Conversely, the XRCC1 194 Arg/Trp polymorphism exhibits a protective effect in women with cervical or endometrial cancer undergoing radiotherapy (67). Furthermore, gene silencing of XRCC2 leads to diminished repair of radiation-induced cellular damage, resulting in G2/M phase arrest and enhanced apoptosis (68). Additionally, the XRCC3 IVS5-14 polymorphic allele is significantly correlated with an elevated risk of late radiotherapy reactions, including fibrosis (67).

### 2.3 Epigenetics and genetic variations

Accumulating evidence highlights the pivotal roles of radiation-induced epigenetic regulation and biological processes in the pathogenesis and progression of RIII. The epigenetic system encompasses alterations in DNA methylation and histone modifications, enabling heritable gene silencing without modifying the underlying coding sequences. The development of “epigenetic therapies” shows considerable promise, particularly through the use of inhibitors targeting enzymes that regulate epigenetic modifications, such as DNA methyltransferases (DNMTs) and histone deacetylases (HDACs), which have exhibited promising radioprotective effects (69). Moreover, the reversibility of these processes and their involvement of multiple dysregulated enzymes suggest that inhibiting histone-modifying enzyme activity and modulating their levels represent viable therapeutic strategies for mitigating RIII.

IR exposure induces DNA methylation modifications, which in turn alter and regulate the expression of associated genes and proteins. This process runs concurrently with classical biological responses to radiation, ultimately resulting in tissue and organ damage (70). However, the expression of DNMTs in response to radiation exposure is influenced by various factors, including cell type, radiation dose, tissue type, and organism sex (71). To date, there is a lack of relevant research on the role of DNMTs in radiation-induced enteropathy; hence, this review does not address DNA methylation in this context. The subsequent sections elucidate the fundamental mechanisms of histone modifications and other critical protein post-translational modifications (PTMs), as well as their roles in RIII.

#### 2.3.1 Histone modifications

Radiation acutely inhibits histone acetylation, leading to chromatin condensation and transcriptional repression. Multiple studies (72, 73) have demonstrated that histone deacetylase inhibitors (HDACi) exert mitigating effects on RIII.

In experimental studies (72) examining the protective efficacy of 7,8-diacetyloxy-4-methylthiocoumarin (DAMTC) against RIII in murine models subjected to total body irradiation (TBI), DAMTC administration at 24 h post-TBI activated anti-apoptotic signaling pathways mediated by the Bcl-2 protein family, consequently mitigating apoptosis in intestinal crypt progenitor/stem cells (ICPS) and villus stromal cells. This intervention facilitated both structural reconstruction and functional restoration of absorptive capacity. Furthermore, DAMTC attenuated TBI-induced DNA damage accumulation, suppressed cell cycle arrest, and conferred radioprotection to ICPS cells through the synergistic activation of anti-P53 and anti-P21 signaling pathways. Additionally, DAMTC enhanced crypt stem cell proliferation, upregulated antioxidant defense mechanisms, inhibited TBI-induced lipid peroxidation, and induced polarization of intestinal macrophages toward anti-inflammatory M2 phenotypes. These findings collectively demonstrate that DAMTC ameliorates RIII through epigenetic regulation via protein acetylation, initiating anti-apoptotic and pro-survival pathways that promote ICPS cell proliferation and maintenance.

In a distinct investigation (73) conducted on 10–12-week-old male C57BL/6 mice, 15 Gy gamma radiation was delivered to the whole abdomen, followed by intravenous trichostatin A (TSA) (150 ng/kg) administration after 1 h and 24 h. It found that the histone deacetylase inhibitor (HDACi) TSA significantly attenuated γ-histone 2AX(H2AX) expression and reduced poly ADP-ribose polymerase 1 (PARP1) and XRCC1 levels in the epithelial cells of the jejunum, suggesting its efficacy in mitigating radiation-induced DNA damage through the regulation of DNA repair mechanisms. Notably, through immunoblotting and qRT-PCR analysis, it was found that TSA administration restored β-catenin expression, decreased dickkopf-related protein 1(DKK1) and glycogen synthase kinase 3 β (GSK3β) levels, and substantially recovered Lgr5 and Bmi1 expression in the murine subjects. Additionally, intestinal morphology exhibited significant enhancement, with restoration of villus height and villus/crypt depth ratios. The research indicates that TSA ameliorates both acute and delayed radiation-induced intestinal damage by modulating the Wnt/β-catenin and TGFβ/Smad signaling pathways, thereby facilitating intestinal stem cell proliferation and regeneration.

What’s more, in another separate murine experiment involving 15Gy abdominal irradiation, TSA alleviated the oxidative-reductive imbalance induced by IR via the NRF2/GPX4/PINK1/PARKIN signaling pathway and reduced apoptosis in intestinal epithelial cells (74).Collectively, these findings (73, 74) underscore the therapeutic potential of HDACi-mediated histone acetylation activation as an innovative strategy for radiation protection. Considering the established clinical applications of HDACi in cancer therapy, the enhancement of protein acetylation may represent a promising avenue.

#### 2.3.2 Non-histone protein modifications

Beyond histone modifications, other pivotal protein modifications, including phosphorylation, acetylation, and ubiquitination, play crucial roles in regulating cellular apoptosis, DNA repair, inflammatory responses, and intestinal barrier function to confer protective effects. The underlying mechanisms and recent research advancements are elaborated as follows (75, 76).

##### 2.3.2.1 Phosphorylation

Following radiation exposure, key proteins such as ATM kinase, P53, and NRF2 undergo phosphorylation and subsequent activation, initiating downstream signaling pathways that promote DNA repair or induce cell cycle arrest, thereby mitigating oxidative stress-induced damage. The specific roles of these proteins have been extensively discussed in preceding section

##### 2.3.2.2 Acetylation

P53 Lys382 acetylation: The acetylation of lysine 382 by CBP/p300 enhances P53’s DNA-binding affinity, thereby upregulating P21 expression to facilitate DNA repair (75). Future investigations may explore the application of acetylation mimetics to amplify P53 functionality and reduce apoptosis in intestinal cells.

##### 2.3.2.3 Ubiquitination

The proteasome inhibitor bortezomib impedes the degradation of ubiquitinated P53 proteins, thereby stabilizing P53 and simulating a persistent stress state (76).

### 2.4 Chronic fibrosis mechanisms

Although acute radiation injury is generally reversible, chronic radiation-induced intestinal damage is characterized by progressive fibrosis, which can lead to long-term complications such as stricture formation, fistulae, and impaired intestinal function. In the context of chronic fibrosis, myofibroblasts act as the primary effector cells, driving structural remodeling through collagen secretion and extracellular matrix (ECM) deposition (6). Following radiation exposure, TGF-β1 activates connective tissue growth factor (CTGF) via the Smad3/4 signaling pathway, thereby promoting collagen synthesis and myofibroblast differentiation (77). As a downstream effector, CTGF is regulated by the Rho/ROCK pathway, which inhibits NF-κB activity (78). Platelet-derived growth factor-BB (PDGF-BB) enhances fibroblast proliferation and chemotaxis through tyrosine kinase receptors, synergizing with TGF-β1 to exacerbate fibrosis (79).

Reserve intestinal stem cells (rISCs) are activated in response to injury and contribute to tissue repair through the Wnt/β-catenin and Notch signaling pathways. Within 24 h post-radiation exposure, β-catenin undergoes nuclear translocation, thereby enhancing Lgr5+ cell proliferation (80) sustain stemness and facilitate tissue regeneration. Nevertheless, negative regulators of the Wnt-β-catenin pathway (DKK1, SFRP4) demonstrate significant upregulation by 72 h, leading to impaired crypt regeneration. BCN057, a canonical Wnt-β-catenin signaling agonist, enhances crypt formation efficiency threefold following radiation exposure (81). Under physiological conditions, BMP signaling counteracts Wnt activity to prevent crypt hyperproliferation and maintain villus-crypt gradients. BMPR1A-deficient murine models exhibit accelerated crypt regeneration post-radiation (82). Notch signaling synergizes with Wnt to promote secretory cell differentiation (e.g., goblet cells, Paneth cells), thereby preserving crypt architecture; however, excessive activation results in ISC depletion. γ-secretase inhibitors (e.g., DAPT) induce a 48-h delay in crypt regeneration (83).

Furthermore, epidermal growth factor (EGF) signaling plays a pivotal role in intestinal cell proliferation, tissue repair, and radioprotection. IR exposure stimulates IL-33 production by ISCs, thereby accelerating epithelial regeneration through EGFR-mediated upregulation of EGF in Paneth cells (78). IL-22 significantly enhances crypt cell survival following radiation exposure and augments organoid formation capacity in a dose-dependent manner, with its protective effects persisting for up to 7 days (84). Exogenous heparin-binding (HB)-EGF, a member of the EGF family, exhibits potent crypt-proliferative and radioprotective properties.

Gene expression profiling elucidates radiation-induced molecular alterations associated with macrophage polarization. Key mediators including TNFα, IL-6, IL-10, chemokine receptor 7 (Ccr7), heme oxygenase 1 (Hmox1), and IL-1β have been identified as crucial factors in M1/M2 polarization, with distinct expression patterns observed across macrophage subsets (85). Conventionally, M1 macrophages have been characterized as fibrinolytic cells due to their secretion of proteolytic enzymes, particularly matrix metalloproteinases (MMPs) and cathepsin K, which facilitate collagen degradation (86, 87). However, emerging evidence indicates that M1 macrophages also possess pro-fibrotic capabilities (88–90). The chronic activation of M1 pro-inflammatory signaling pathways, coupled with persistent and ineffective injury-repair cycles, ultimately results in tissue fibrosis and structural remodelin (78).

In addition, Bessout et al. (21) demonstrated that Th17 cells play a predominant role in radiation-induced colonic injury. Their experimental findings revealed that incubation of irradiated colonic smooth muscle cells with recombinant IL-17 protein significantly upregulates the expression of IL-6 and IL-1β, along with genes associated with extracellular matrix remodeling. Beyond its interaction with stromal cells through MAPK or NF-κB signaling pathways, IL-17 also contributes to fibrotic processes by facilitating neutrophil recruitment and augmenting neutrophil elastase activity (91).

### 2.5 Role of gut microbiota dysbiosis

Contemporary research demonstrates that IR instigates gut microbiota dysbiosis, fundamentally transforming its structural configuration, functional dynamics, and species diversity, while simultaneously modifying its metabolic profile (92, 93). The Firmicutes/Bacteroidetes (F/B) ratio is crucial for maintaining normal intestinal homeostasis. An increase or decrease in the F/B ratio is considered a sign of dysbiosis, with the former typically associated with obesity and the latter with inflammatory bowel disease (94). These alterations potentially exacerbate IR-induced tissue damage and amplify pro-inflammatory immune responses. In contrast, symbiotic microorganisms and their beneficial metabolic byproducts can reconstitute the disrupted intestinal microbial architecture during IR exposure, facilitate the restoration of homeostasis between anti-inflammatory and pro-inflammatory mechanisms, and consequently attenuate RIII (95).

In different mouse models that all received 18Gy radiation to the abdomen, the F/B ratio could increase or decrease (96, 97). Only the F/B ratio of the soluble gp130 transgenic mice exhibited a meaningful postirradiation decrease, whereas the F/B ratios of Galectin-1 KO mice and wild-type mice increased after irradiation (96). Notably, in radiation-resistant murine strains exhibiting prolonged survival post high-dose irradiation, the enhanced proliferation of *Lachnospiraceae* and *Enterococcaceae* families demonstrated a positive correlation with hematopoietic reconstitution and gastrointestinal mucosal repair, accompanied by elevated fecal concentrations of propionate and tryptophan-derived metabolites (98).

Following IR in non-human primates (NHPs; *Macaca mulatta*), the intestinal microbiota composition undergoes substantial modifications. The F/B ratio exhibited a marked decline from 1.2 to below 1.0 subsequent to 7.4 Gy radiation exposure. Of particular significance, the relative abundances of *Actinobacteria*, *Fusobacterium*, *Prevotella*, and *Veillonella* demonstrated a more than twofold increase, whereas *Aerobacter* and *Acinetobacte*r populations experienced a remarkable reduction exceeding tenfold (99).

In clinical studies, radiotherapy targeting abdominal or pelvic tumors has been shown to significantly decrease the F/B ratio in 11 patients (100) and significantly reduce α-diversity of gut microbiota, while concurrently increasing the prevalence of pathogenic bacteria (e.g., *Enterobacteriaceae*, *Escherichia coli*) and decreasing beneficial microbial taxa (e.g., *Bacteroides*) (95). A longitudinal clinical cohort study has revealed a progressive decline in microbial diversity corresponding to the advancement of RIII (4). Furthermore, a robust correlation has been established between diminished bacterial diversity and the progression of RIII. Notably, elevated abundances of *Clostridium cluster IV*, *Rothia*, and *Phascolarctobacterium* demonstrate significant associations with the severity grading of RIII.

Thus, it can be seen that the changes in F/B in different models of RIII are not characteristic. The specific reasons need further research. However, in general, all studies have shown that exposure to IR leads to a decrease in the diversity and richness of the biological community, an increase in the abundance of pathogenic bacteria (Proteobacteria and Fusobacteria), and a reduction in beneficial bacteria (*Faecalibacterium* and *Bifidobacterium*) (92, 97). In the context of dynamic changes in the F/B ratio, the dynamic characteristics of the microbial composition of the two main bacterial groups and other components largely reflect the dysregulation of the intestinal microbiota during the occurrence and development of RIII.

Gut microbiota dysbiosis exacerbates RIII through the following mechanistic pathways (95):

a. Disruption of biological barriers: Pathogenic bacterial species (e.g., *Escherichia coli*) enzymatically degrade secretory immunoglobulin A (sIgA), thereby compromising the integrity of intestinal immune defenses.
b. Dysregulation of immune homeostasis: Lipopolysaccharide (LPS)-positive bacteria trigger the activation of proinflammatory cytokine cascades through Toll-like receptor 4 (TLR4)/NF-κB signaling pathways.
c. Suppression of anti-inflammatory mediators: The diminished production of probiotic-derived metabolites, particularly short-chain fatty acids (SCFAs) including butyrate, leads to reduced IL-10 activity.

## 3 Diagnosis and biomarkers

### 3.1 Imaging modalities

Currently, there are no clear diagnostic criteria for radiation-induced intestinal injury. In clinical practice, modern cross-sectional imaging techniques such as CT and MRE have become the preferred diagnostic tools due to their high resolution and non-invasive advantages (101, 102). CT provides high-resolution anatomical images through X-ray tomography, with radiation doses lower than traditional X-rays (total exposure <1 mSv) (103). It exhibits high sensitivity for detecting tumors, inflammation, and intestinal obstruction, particularly in emergency settings.

MRE, based on magnetic resonance imaging (MRI) principles, uses oral contrast agents to distend the intestines and generate high-resolution soft-tissue images via magnetic fields and radiofrequency pulses. This non-IR feature is a significant advantage for patients who have previously undergone radiotherapy. Additionally, MRE offers superior soft-tissue contrast resolution compared to CT, enabling clearer visualization of intestinal wall inflammation, fibrosis, and thickening (104, 105). Theoretically, T2-weighted imaging (T2WI) can clearly depict intestinal wall edema (high signal) and fibrosis (low signal), differentiating acute inflammation (predominantly edematous) from chronic injury (predominantly fibrotic). Diffusion-weighted imaging (DWI), on the other hand, quantifies inflammation activity by measuring the degree of water molecule diffusion restriction, aiding in the early detection of radiation-induced intestinal damage (106). This distinction is critical for guiding treatment decisions. Although we have not yet collected any real-world clinical data to validate this hypothesis, we believe that MRE could potentially emerge as one of the important modalities for diagnosing radiation-induced intestinal injury in the future. Nevertheless, the choice between MRE and CT often depends on institutional resources, patient tolerance, and specific clinical scenarios.

### 3.2 Endoscopic innovations: confocal laser endomicroscopy

The advancement of endoscopic technology has given rise to confocal laser endomicroscopy (CLE) as a promising tool for real-time, *in vivo* assessment of intestinal diseases. CLE is an endoscopic technique capable of obtaining ultra-high magnification images of the gastrointestinal mucosa (8), with the potential to enable real-time histological diagnosis (107). Its principle relies on low-power laser illumination of tissue and the subsequent detection of fluorescent light reflected back from the tissue through a pinhole (108). The term “confocal” refers to the alignment of both the illumination and collection systems within the same focal plane (109, 110). This design significantly enhances spatial resolution, permitting real-time cellular imaging and tissue architectural evaluation during endoscopy (8), thereby providing detailed insights into epithelial integrity, vascular changes, and inflammatory infiltration.

Currently, this technology has been extensively applied to characterize morphological features in inflammatory bowel disease (IBD) (111) and has also been utilized for microscopic mucosal descriptions in irritable bowel syndrome (IBS), infectious colitis (e.g., *Clostridioides difficile* infection) (112), and colonitis associated with graft-versus-host disease (GVHD) (111). Although the widespread adoption of CLE is limited by its high cost, steep learning curve, and requirement for specialized equipment, we propose that it could serve as a safe and feasible diagnostic tool for radiation-induced intestinal injury. Specifically, CLE may aid in assessing intestinal morphology, evaluating disease activity, and predicting treatment responses in this context.

### 3.3 Innovative approaches in biomarkers

MicroRNAs (miRNAs) are small non-coding RNAs involved in post-transcriptional regulation. Due to their stability in body fluids (e.g., serum, urine) and radiation sensitivity, they serve as promising candidate biomarkers for radiation exposure (19). Numerous studies have identified tissue-specific miRNA signatures associated with radiation exposure time and dose in animal models and patients. Rogers, C. J. et al. discovered modified miRNA profiles following radiation exposure and demonstrated that a miRNA panel could predict risks of neutropenia and pulmonary fibrosis in non-human primate models, with partial clinical validation in human samples (113). In intestinal tissues, radiation induces gene enrichment related to cell cycle regulation and metabolic processes. In irradiated mouse intestines, significant changes were observed in miR-34a and miR-215, which regulate cell cycle progression and DNA repair, thereby influencing intestinal mucosal repair post-radiation (19). These miRNAs may represent potential specific biomarkers for radioactive enteritis. However, future clinical translation must address challenges such as cross-species differences and dynamic complexity.

Fatty acid binding proteins (FABPs) are a class of low-molecular-weight cytosolic proteins widely expressed in intestinal epithelial cells. They are rapidly released into the bloodstream during tissue ischemia, oxidative stress, or inflammation, possessing significant potential as biomarkers for injury. Suchitra Sharma and colleagues (114) employed a data mining approach to retrieve six microarray datasets from the NCBI Gene Expression Omnibus (GEO) database and identified differentially expressed FABP genes across species. Subsequently, quantitative reverse transcription polymerase chain reaction (qRT-PCR) was used to analyze the abundance of ten FABP-encoding genes in the intestinal tissues of male and female C57Bl/6 mice. The study revealed that FABP1 and FABP2 are highly expressed genes in the small intestine of both sexes. If elevated levels of FABP1/FABP2 are observed in the blood of humans exposed to radiation, rapid diagnostic assays (e.g., ELISA) could be developed to aid in early diagnosis.

Over time, circulating EVs have also been studied as biomarkers in liquid biopsies due to their high stability and presence in all body fluids. Exosomes are nanoscale vesicles secreted by cells, rich in proteins, lipids, and nucleic acids. By isolating exosomes from plasma and conducting metabolomic/lipidomic analyses, low-abundance biomarkers that are difficult to detect in traditional plasma analyses can be captured (115). Current research has focused on exosomes for studying radiation therapy complications. Institut de Radioprotection et de Sûreté Nucléaire (IRSN) identified protein components in EVs associated with coagulation and inflammatory processes in prostate cancer patients undergoing radiation therapy (116). A multicenter prospective study is exploring early detection of cardiac complications after breast cancer radiotherapy, with preliminary results showing dose-dependent increases in platelet- and endothelial-derived EVs (117). Further studies are needed to validate the role of exosomes in the early diagnosis of RIII.

The diversity of the gut microbiome is closely linked to radiation sensitivity, with shifts in microbial composition serving as early warning indicators of radiation-induced damage (100). Studies (19) have found that a sharp decline in *Lactobacillus* abundance in feces correlates negatively with the severity of RIII, while the proliferation of pathogenic bacteria (e.g., *Clostridium perfringens)* is associated with an increased risk of delayed intestinal fibrosis. Simply put, radiation exposure leads to a reduction in “beneficial bacteria” and an overgrowth of “pathogenic bacteria.” However, individual responses to radiation-induced microbiome changes vary significantly, potentially influenced by genetic factors, diet, baseline gut microbiota composition, and other variables. Future efforts should focus on developing machine learning-based predictive models that integrate multi-omics data (e.g., metagenomics, metabolomics) to enhance the precision of personalized interventions.

In conclusion, the diagnosis of RIII is evolving with the integration of advanced imaging techniques, innovative endoscopic tools, and novel biomarkers. These advancements are paving the way for more accurate and timely diagnosis, ultimately improving patient outcomes in this challenging condition.

## 4 Treatment strategies

The pathological process of RIII involves multi-layered molecular and cellular events. From acute-phase DNA damage and intestinal inflammation to chronic-phase fibrosis and microcirculation dysfunction, each stage presents potential therapeutic targets. Ongoing research into the mechanisms of RIII has revealed that certain novel drugs and treatment strategies exhibit significant therapeutic potential for RIII. Furthermore, current drug development efforts focus on creating Radioprotectors (for pre-radiation use) and Radiomitigators (for post-radiation use) (19). Radioprotectors are administered prior to radiation exposure as preventive agents to inhibit or minimize radiotherapy-induced adverse effects through mechanisms such as free radical scavenging and DNA damage prevention. In contrast, radiomitigators are employed post-radiation exposure as reactive agents to alleviate established radiation-related complications by modulating inflammatory cascades and promoting cellular repair (19). Therefore, the tables include corresponding categories. It should be noted that if RIII progresses to irreversible chronic ischemic necrosis and fibrosis of the intestinal wall, surgical intervention would be the most ideal treatment option (1, 118). The surgical treatment methods are not discussed in detail in this article. In this section, we review potential therapeutic strategies for RIII based on preclinical and clinical trials. But the content related to clinical trials will be uniformly presented in the next chapter.

### 4.1 Natural plants and their derivatives

Studies have demonstrated that natural plants and their derivatives exhibit pharmacological activity and play a significant role in RIII (Table 1). Most of them mitigated RIII via improving intestinal barrier, inhibiting oxidation, regulating gut microbiota, decreasing inflammation, restricting apoptosis, promoting intestinal stem regeneration,etc.

**TABLE 1:** Natural Plants and their Derivatives.

Therapy	Uses	Model	Action	References
RK1	Radiomitigator	Sprague‒Dawley rats; IEC-6	Regulated gut microbiota, inhibited oxidation and restricted apoptosis	(122)
Resveratrol	Radioprotector and Radiomitigator	IEC-6	Inducted autophagy, inhibited oxidation and restricted apoptosis	(123, 124)
Baicalein	Radioprotector and Radiomitigator	C57BL/6 mice	Repaired endothelial injury, regulated gut microbiota, decreased inflammation and improved intestinal barrier	(125, 126)
EGCG	Radioprotector and Radiomitigator	C57BL/6 mice; HIEC	Regulated gut microbiota, promoted stem regeneration and inhibited apoptosis and ferroptosis	(2, 127)
TFERL	Radioprotector and Radiomitigator	C57BL/6 mice; HIEC-6	Inhibited oxidation, reduced DNA damage, restricted apoptosis and ferroptosis	(128)
Zingerone derivate	Radioprotector and Radiomitigator	C57BL/6 mice	Inhibited apoptosis and promoted the proliferation and differentiation of crypt cells	(129)
Apigenin	Radioprotector and Radiomitigator	C57BL/6 mice	Inhibited oxidation, restricted apoptosis, and promoted intestinal epithelial regeneration	(130)
Ecliptae Herba	Radioprotector and Radiomitigator	Sprague‒Dawley rats; C57BL/6 mice	Inhibited oxidation, restricted apoptosis, maintain the stability of cell cycle, and promoted intestinal stem regeneration	(131)
Lycium barbarum	Radiomitigator	C57BL/6 mice; IEC-6	Immunomodulated and the synergistically modulated effect on the gut microbiota and related metabolites	(22)
Paeoniflorin	Radioprotector	RAW264.7 cells; HUVEC	Improved intestinal barrier, and decreased inflammation	(132)
Procyanidin B2	Radioprotector	C57BL/6 mice	Inhibited Oxidation, and promote intestinal stem regeneration	(133)
DIM	Radiomitigator	C57BL/6 mice; HIEC-6	Inhibited oxidation, restricted apoptosis, repaired DNA damage, and regulated gut microbiota	(134)
Sanguinarine	Radioprotector	C57BL/6 mice; IEC-6	Regulated gut microbiota	(135)
Chamomile extract	Radioprotector and Radiomitigator	Wistar rats	Inhibited oxidation, restricted apoptosis, and decreased inflammation	(136)
TT-2	Radioprotector and Radiomitigator	IEC-6	Promoted the regeneration of intestinal epithelial cells and stem cells	(137)
HSP1	Radioprotector and Radiomitigator	C57BL/6 mice	Improved intestinal barrier, inhibited oxidation, and regulated gut microbiota	(138)
Isoliquiritigenin	Radiomitigator	C57BL/6 mice	Decreased inflammation, restricted apoptosis, promoted intestinal stem regeneration, and regulate gut microbiota	(139)

RK1: Ginsenoside Rk1; EGCG: (−)-Epigallocatechin-3-Gallate; TFERL: total flavonoids of Engelhardia roxburghiana Wall. Leaves; DIM: 3,3′-Diindolylmethane; TT-2: an active fraction of the rhizomes of Trillium tschonoskii Maxim; HSP1: one water-soluble polysaccharides from *H. sabdariffa*.; IEC: intestine epithelial cell; HIEC: human intestinal epithelial cell; HUVEC: human umbilical vein endothelial cells.

Ginsenosides, extracted from Panax ginseng through thermal processing, are recognized as the primary bioactive components of ginseng. Ginsenoside Rk1 (RK1) (119), a rare ginsenoside, exhibits multiple bioactivities including anticancer effects, anti-apoptotic properties, anti-inflammatory responses, antioxidant capacity and antiplatelet aggregation effects. Accumulating evidence indicates that ginseng and its purified components containing RK1 demonstrate radioprotective potential by attenuating radiation-induced injury through oxidative stress reduction mechanisms (120, 121). Yilin Wang et al. (122) discovered that RK1 treatment significantly enhanced the survival rate of irradiated rats and significantly improved the intestinal mucosal structure damage observed through histological examination. RK1 treatment significantly alleviated the oxidative stress-induced apoptosis of intestinal epithelial cells induced by radiation. In addition, RNA-Seq identified 388 differentially expressed genes (DEGs), and indicated that the PI3K-AKT pathway might be the key signaling pathway for RK1 to exert therapeutic effects on RIII. Western blotting results showed that the expression levels of p-PI3K, p-AKT, and p-mTOR increased due to radiation were significantly inhibited by RK1.

Coincidentally, in another study on resveratrol (123), the PI3K/AKT/mTOR signaling pathway was also mentioned. Intestine epithelial cells (IEC)-6 were pretreated with resveratrol 2 h before radiation exposure. It was observed that resveratrol alleviated radiation-induced oxidative stress and cellular damage by activating autophagy through inhibition of the PI3K/AKT/mTOR signaling pathway, and upregulated SIRT1 expression post-irradiation, achieving an anti-apoptotic effect.

Secondly, for the treatment of RIII, protecting endothelial cells is of great significance. Baicalin is one of the natural plants and their derivatives which can affect endothelial cell function. Hyosun Jang et al. (125) observed that villi length was shortened, and intestinal crypt function was impaired in radiation-induced colitis mouse models. After treatment with baicalin, intestinal damage was alleviated, and intestinal barrier function was improved. Baicalin inhibited the expression of radiation-induced adhesion molecules in human umbilical vein endothelial cells (HUVECs) and in the intestines of irradiated mice, and reduced leukocyte infiltration in radiation-induced colitis.

Moreover, Meifang Wang et al. (126) also discovered that baicalein can re-adjust the composition pattern of intestinal microorganisms that has been disrupted by irradiation, increase the relative abundance ratio of Bacteroidetes and Firmicutes, and reduce the level of Proteobacteria. At the same time, baicalein inhibited the activation of P53 and mediated mitochondrial apoptosis and death receptor apoptosis in the intestine through P53. Eventually, it improved the intestinal structure, promoted the proliferation and regeneration ability of intestinal cells.

As for the regulation of gut microbiota, it is also a common mechanism of action for various drugs. Regulation of gut microbiota was also discovered in *Lycium barbarum* (22), *Isoliquiritigenin* (139), One water-soluble polysaccharides from *H. sabdariffa* (HSP1) (138), (−)-Epigallocatechin-3-Gallate (EGCG) (127), etc…

However, Li-Wei Xie et al. (2) conducted further research and discovered that in human intestinal epithelial HIEC cells, the same radiation protection effect was observed as in mice, manifested as a reduction in the number of γH2AX foci and a decrease in ferroptosis. In addition, EGCG lowered the level of ROS and activated transcription factor NRF2 and its downstream targets, including antioxidant proteins Slc7A11, HO-1, and GPX4. The use of NRF2 inhibitor ML385 eliminated the protective effect of EGCG, indicating that the activation of NRF2 is essential for the activity of EGCG. Therefore, EGCG can also counteract RIII by eliminating ROS and inhibiting apoptosis and ferroptosis.

Inhibition of ferroptosis can also be discovered in total flavonoids of Engelhardia roxburghiana Wall. leaves (TFERL). Seven days before and 7 days after the irradiation at 7.2 Gy, C57BL/6 mice were administered with different doses of TFERL (20, 40, and 80 mg/kg), and the researchers (128) found that TFERL alleviated RIII by reducing the damage of intestinal crypt/villus structure, increasing the number and proliferation of ISCs, and maintaining the integrity of intestinal epithelium after abdominal irradiation. Moreover, TFERL promoted the proliferation of irradiated HIEC-6 cells and reduced radiation-induced cell apoptosis and DNA damage. Mechanistic studies showed that TFERL promoted the expression of NRF2 and its downstream antioxidant proteins, and silencing NRF2 led to the loss of TFERL’s radiation protection effect, indicating that TFERL exerted radiation protection through activating the NRF2 pathway.

### 4.2 Traditional Chinese medicine (TCM)

TCM represents a comprehensive diagnostic, pathophysiological, and therapeutic system rooted in over two millennia of accumulated knowledge and clinical practice. This system encompasses herbal medicine, acupuncture, and various physical therapeutic modalities (140). Empirical studies have demonstrated that TCM exhibits significant protective effects against RIII. Chinese herbal decoction is a traditional form of herbal medicine in TCM, made by boiling a combination of dried herbs, roots, barks, seeds, or flowers in water. It is one of the most common and classic ways to administer herbal remedies, valued for its potency and ability to target specific health issues. In the following text, we list some traditional Chinese herbal decoction that have been studied for the treatment of RIII.

In accordance with the principles of TCM and through an integrated approach combining network pharmacology analysis with experimental validation, researchers (141) have identified that Liangxue Guyuan Yishen Decoction (LGYD) significantly enhances the survival rate o RIII model rats, mitigates radiation-induced intestinal pathological damage, and facilitates intestinal crypt regeneration. The investigation revealed that LGYD upregulates the expression of LGR5+ intestinal stem cell markers, stimulates the proliferation of crypt stem cells, augments tight junction protein expression, and restores intestinal epithelial barrier function. Through network pharmacology predictions and experimental validations, the study delineated the key signaling pathways underlying its mechanism: the Wnt signaling pathway was significantly activated (as evidenced by targets including β-catenin and c-Myc), while the MEK/ERK pathway was implicated in the regulation of cell proliferation and survival (demonstrated by alterations in ERK phosphorylation levels).

Liying Zhang et al. (142) demonstrated that Guiqi Baizhu Decoction (GQBZD) effectively mitigates immune impairment and intestinal microbiota dysbiosis induced by IR. In experimental studies utilizing rat models, the researchers observed that GQBZD significantly attenuated radiation-induced weight loss, ameliorated reductions in food and water intake, alleviated diarrhea, and improved quality of life scores following X-ray exposure. Furthermore, GQBZD exhibited notable anti-inflammatory properties and enhanced immune function. The formulation restored both α diversity (species richness) and β diversity (community structure) of the intestinal microbiota, thereby counteracting radiation-induced microbial imbalance. Notably, GQBZD reduced the relative abundance of *Desulfovibrio*, *Bacteroides*, and *Parabacteroides* (with the exception of *Roseburia* and a specific genus within *Lachnoclostridium*), indicating its potential protective effects on beneficial bacteria, particularly butyrate-producing species. The treatment also facilitated the production of short-chain fatty acids (SCFAs), reinforced intestinal barrier integrity, and suppressed the phosphorylation of p-P65, a critical component in the NF-κB signaling pathway, consequently reducing inflammatory signaling activation.

In parallel investigations, Yangyang L et al. (143) reported that Sprague-Dawley (SD) rats exposed to 6Gy X-ray irradiation exhibited marked colonic edema accompanied by ultrastructural damage to nuclear and mitochondrial components, increased expression of ROS, hypoxia-inducible factor 1-alpha (HIF-1α), and aquaporin-4 (AQP4), along with decreased expression and activity of Na+/K+-ATPase. However, GQBZD intervention effectively ameliorated colonic edema through the mitigation of hypoxia and oxidative stress, coupled with the regulation of AQP4 and Na+/K+-ATPase expression.

In the experimental investigation utilizing SD rats subjected to abdominal radiotherapy (144), compound kushen injection (CKI) was demonstrated to effectively mitigate radiation-induced intestinal inflammation, ulcerative lesions, and associated clinical manifestations while concurrently ameliorating intestinal tissue damage. Furthermore, CKI administration significantly reduced the concentrations of IL-1β, IL-6, and myeloperoxidase (MPO) within the intestinal mucosa, thereby exerting potent anti-inflammatory effects. Histopathological evaluations revealed that CKI treatment markedly decreased the apoptotic cell count in crypt regions, indicating its inhibitory capacity against epithelial cell apoptosis.

Shaoyao Decoction (SYD), a classical heat-clearing formula, is recognized for its efficacy in purging visceral real heat and is commonly employed in the treatment of various intestinal discomfort symptoms. Clinical evidence indicates its potential therapeutic effects on radiation enteritis. An X-ray-induced RIII model was established using C57BL/6 mice. Histopathological analysis revealed that SYD attenuated epithelial cell shedding and necrosis in the mucosal crypt layer while suppressing abnormal proliferation of damaged fibrous tissue. SYD significantly reduced serum levels of MDA, COX, LPS, and pro-inflammatory cytokines (IL-6, IL-1β, TNF-α), while enhancing the activity of the antioxidant enzyme SOD. TUNEL staining demonstrated that SYD decreased colon cell apoptosis; Western blot and qRT-PCR analyses confirmed that SYD upregulated the expression of Dclk-1, ATM, and MRE-11 genes and proteins, promoted the release of mitochondrial pro-apoptotic proteins Bax, Caspase-3, and Cyto-c, and concurrently increased the level of anti-apoptotic protein Bcl-2. Immunofluorescence analysis showed that SYD significantly reduced P53 protein expression and reversed the downregulation of Claudin-1 protein. These findings demonstrate that SYD effectively ameliorates X-ray-induced radiation enteritis in mice through multiple mechanisms, including tissue fibrosis reduction, inflammatory response inhibition, and regulation of the mitochondrial apoptotic pathway (pro-apoptotic and anti-apoptotic protein balance). The underlying mechanism may involve the regulation of DNA damage repair-related genes (Dclk-1, ATM, and MRE-11) and the restoration of intestinal barrier function mediated by P53 and Claudin-1 (145).

### 4.3 Other compounds and biomolecules

In addition to the aforementioned plant-derived drugs and traditional Chinese medicine formulations, numerous other significant compounds and biomolecules (Table 2) have been extensively validated for their efficacy in regulating RIII both prophylactically and therapeutically. Notably, this includes reusable therapeutic agents which have demonstrated substantial potential in clinical applications.

**TABLE 2:** Other compounds and biomolecules.

Therapy	Uses	Model	Action	References
NAC	Radioprotector	Sprague‒Dawley rats; C57BL/6 mice	Promoted intestinal epithelial regeneration, and improved intestinal barrier	(146, 147)
BCN057	Radiomitigator	C57BL/6 mice	Promoted intestinal epithelial regeneration	(81)
WR-2721-PGN combination	Radiomitigator	C57BL/6 mice	Promoted intestinal epithelial regeneration	(148)
α-Lipoic acid	Radioprotector	C57BL/6 mice	Inhibited oxidation	(149)
PHDs	Radioprotector	C57BL/6 mice	Increased hypoxia-inducible factor expression	(150)
α-tocopherol	Radioprotector	C57BL/6 mice	Inhibited oxidation	(151)
Ex-RAD	Radioprotector	C57BL/6 mice	Restricted apoptosis	(152)
FGF-2 peptide	Radioprotector	C57BL/6 mice	Promoted the regeneration of intestinal crypt cells, and decreased inflammation	(153)
Anti-ceramide antibody	Radioprotector and Radiomitigator	C57BL/6 mice	Restricted apoptosis of endothelial cell	(154, 155)
JNJ-26366821	Radioprotector	C57BL/6 mice	Stimulated hematopoiesis	(156)
OTP	Radioprotector and Radiomitigator	C57BL/6 mice	Stimulated hematopoiesis	(157)
Amifostine	Radioprotector	C57BL/6 mice; BALB/c-Nude mice; NHP; Human	Improved intestinal barrier, inhibited oxidation, and restricted ferroptosis.	(158–161)
Pentoxifylline-vitamin E combination	Radiomitigator	Human	Anti-fibrosis	(162)
GT3	Radioprotector	C57BL/6 mice; NHP	Inhibited oxidation and regulated immune	(163–165)
Recombinant human interleukin-12	Radiomitigator	NHP	Stimulated hematopoiesis and improved intestinal barrier	(166–168)
Interleukin-11	Radioprotector and Radiomitigator	CD2F1 mice	Decreased inflammation and improved intestinal barrier	(169–171)
Entolimod (CBLB502)	Radioprotector and Radiomitigator	C57BL/6 mice; NHP	Restricted apoptosis, promoted the regeneration of intestinal crypt cells, and stimulated cytokine production	(172–174)
OrbeShield™	Radiomitigator	NHP; Minipig	Decreased inflammation, and graduated apoptosis	(175, 176)
ABC294640	Radioprotector	Kaposi’s Sarcoma-like nude mice; Human	Decreased inflammation	(177, 178)
Peptide TP508	Radiomitigator	ICR (CD-1) outbred male mice	Activated radioresistant stem cells	(179)
Auranofin	Radioprotector	C57BL/6, BALB/c and C3H mice; IEC-6	Restricted DNA damage-induced apoptosis	(180, 181)
Metformin	Radiomitigator	C57BL/6 and BALB/c mice	Improved intestinal barrier and regulated gut microbiota	(182, 183)
Pravastatin	Radiomitigator	HIEC; C57BL/6 mice; Gottingen minipigs	Inhibited oxidation, and decreased inflammation	(184, 185)
Rebamipide	Radiomitigator	HT29; C57BL/6 mice; Human	Improved intestinal barrier and promoted the regeneration of intestinal crypt cells	(186, 187)
Ghrelin	Radiomitigator	C57BL/6 mice; B6D21 mice; HIEC	Increased in epithelial proliferation and inhibition of pro-inflammatory cytokine production	(188, 189)
Palifermin	Radioprotector	Human	stimulate the reparative proliferation of epithelial cells	(190, 191)
TSA	Radiomitigator	C57BL/6 mice	Inhibited oxidation, restricted apoptosis and promoted intestinal stem regeneration	(73, 74)

NAC: N-acetylcysteine; BCN057: an anti-neoplastic small molecular agent; WR-2721: 2-(3-aminopropylamino) ethylsulphanyl phosphonic acid; PGN: peptidoglycan, a toll-like receptor 2 agonist; PHDs: Inhibitors of prolyl hydroxylase domain-containing enzymes; Ex-RAD: 4-carboxystyryl-4-chlorobenzylsulfone; FGF-2 peptide: a peptide derived from the receptor binding domain of fibroblast growth factor 2; JNJ-26366821: a PEGylated thrombopoietin mimetic (TPOm) peptide; OTP: Octadecenyl thiophosphate; GT3: Gamma-Tocotrienol; Entolimod (CBLB502): Toll-like receptor 5 agonists; ABC294640: a selective inhibitor targeting sphingosine kinase 2; Peptide TP508: rusalatide acetate, Chrysalin; TSA: Trichostatin A; NHP: Non-human primate; HIEC: Human intestinal epithelial cell line; HT29: Human colonic adenocarcinoma cell line HT29.

Firstly, emerging research has revealed that several established clinical agents demonstrate corresponding therapeutic efficacy in animal or cell models of RIII (Table 2), such as metformin, pravastatin, and auranofin. Metformin, the most extensively utilized hypoglycemic agent in clinical practice, exhibits multifaceted biological properties beyond its glucose-lowering effects, encompassing antioxidant, anti-inflammatory, and anti-apoptotic activities (192, 193). In an investigation into metformin’s impact on RIII, Hyosun Jang et al. conducted abdominal irradiation of mice with 13.5 Gy using X-rad-320, followed by metformin administration. The study (182) revealed that metformin treatment augmented the expression of tight junction proteins in epithelial cells and impeded bacterial translocation to mesenteric lymph nodes. Furthermore, metformin enhanced the population of ISCs by mitigating radiation-induced toxicity and facilitated epithelial cell regeneration through activation of the Wnt/β-catenin signaling pathway. Jing-Yu Yang et al. (183) demonstrated a significant increase in the abundance of lactic acid bacteria in the intestinal microbiota of patients receiving metformin during abdominal radiotherapy, with an inverse correlation observed between bacterial abundance and the duration of diarrhea. Additionally, the combination of metformin with *Lactobacillus* supplementation or FMT was found to activate the Farnesoid X Receptor (FXR) signaling pathway in intestinal epithelial cells, resulting in the upregulation of tight junction proteins and mucins, along with an increase in goblet cell numbers, thereby preserving the integrity of the intestinal epithelial barrier.

Pravastatin, a widely utilized therapeutic agent in clinical settings for the reduction of serum cholesterol levels, has been demonstrated to exhibit anti-inflammatory properties on endothelial cells. Hyosun Jang et al. (184) conducted a comprehensive investigation into the therapeutic efficacy of pravastatin on radiation-induced enteritis-damaged epithelial cells through both *in vitro* and *in vivo* experimental systems. The research findings revealed that pravastatin effectively suppressed radiation-induced intestinal inflammatory cytokines, specifically IL-6, IL-1β, and TNF-α, in irradiated InEpC cells. Furthermore, in murine models subjected to total abdominal irradiation and treated with pravastatin, histological impairments including villous shortening and intestinal crypt dysfunction were significantly ameliorated, with the differentiation of intestinal epithelial cells restored to normal physiological levels. In another investigation employing the minipig model by quantifying clinical symptoms, irradiated minipig demonstrated attenuated clinical manifestations, reduced intestinal inflammatory responses and endotoxin concentrations, and enhanced gastrointestinal mucosal integrity. Concurrently, through mRNA sequencing analysis, Kwak, Seo Young et al. proposed that pravastatin exerts its regulatory effects on epithelial integrity via modulation of MT2 expression in radiation-compromised epithelial cells (185).

Secondly, amifostine stands as the sole small-molecule radioprotective agent that has received approval from the U.S. Food and Drug Administration (FDA) in recent decades. And it has been extensively validated in murine radiation models to restore glutathione metabolism, neutralize free radicals, and exert potent antioxidant effects. This compound effectively suppresses arachidonic acid metabolism, thereby reducing pro-inflammatory mediators and modulating anti-inflammatory responses. Furthermore, amifostine demonstrates significant regulatory effects on bile acid, folate, vitamin A, and amino acid metabolism, facilitating the restoration of metabolic homeostasis. Through its dual mechanisms of supporting crypt stem cell regeneration and accelerating mucosal repair, it provides comprehensive protection for intestinal epithelial cells, ultimately delivering multi-faceted radioprotective effects on the jejunum (160).

Nevertheless, the severe adverse reactions associated with this compound have significantly constrained its clinical utility. To mitigate the toxicity concerns while preserving therapeutic efficacy, a series of cysteine-modified small peptides have been synthesized. Notably, compound 5 demonstrates radioprotective efficacy equivalent to that of amifostine, while exhibiting superior safety profiles. Intriguingly, mechanistic investigations have revealed that compound 5 exerts its cytoprotective effects through the inhibition of IR-induced ferroptosis, a distinct mechanism from that of amifostine (158).

Thirdly, previous studies (194–196) have revealed that the depletion of stem cell clonogens (SCCs) in the crypts of Lieberkühn is closely linked to ceramide-induced endothelial cell apoptosis within the mucosal microvascular network, which transmits apoptotic signals. A monoclonal antibody named 2A2, targeting ceramide, binds to ceramide and prevents the formation of “ceramide-rich platforms” in the endothelial cells of the gastrointestinal tract in irradiated mice (154). In addition, the successful generation of the single-chain variable fragment (scFv) derived from ceramide 6B5 antibody was accomplished, and subsequent experimental validation revealed that administration of 6B5 scFv significantly reduced mortality rates in murine models when administered subcutaneously 24 h post-exposure to a 90% lethal dose (15 Gy) of radiation in the context of RIII (155). This mechanism protects endothelial cells in the small intestinal lamina propria from apoptosis, promotes the recovery of crypt SCCs, and prevents murine mortality from RIII following high-dose radiation exposure.

The PEGylated thrombopoietin mimetic peptide (PEG-TPOm), which has demonstrated efficacy in treating murine hematopoietic acute radiation syndrome, has been unexpectedly found to exhibit vascular protective properties by mitigating hemorrhage and intestinal permeability. Additionally, it stimulates crypt cell proliferation and enhances survival rates when administered following radiation exposure (156).

Similarly, the small molecule octadecenyl thiophosphate (OTP), a synthetic analog of lysophosphatidic acid (LPA), exhibited significant radioprotective and radiomitigative effects in a murine model of total-body irradiation (TBI). A single dose of OTP administered within the time window of 12 h pre-irradiation to 26 h post-irradiation demonstrated a 50% reduction in mortality rate. Notably, even when administered during the 48–72 h post-irradiation period (corresponding to the late phase of acute radiation syndrome), OTP maintained a mortality reduction of ≥34%. Furthermore, OTP treatment enhanced jejunal crypt survival, improved intestinal glucose absorption capacity, reduced systemic endotoxin leakage, and mitigated radiation-induced intestinal injury (157).

Collectively, vascular-targeted therapeutic approaches for RIII, including anti-ceramide antibody administration and thrombopoietin mimetic application, effectively address the constraints inherent in conventional DNA repair-based therapeutic modalities through endothelial cell protection and regenerative promotion, thereby manifesting dual therapeutic efficacy encompassing both acute intervention and tissue restoration.

Lastly, antioxidants, particularly those compounds classified within the vitamin E category, have been extensively investigated and widely utilized as radioprotective agents (197). Gamma-Tocotrienol (GT3), a constituent of the vitamin E family distinguished by its potent antioxidant properties and inhibitory action on 3-hydroxy-3-methylglutaryl-coenzyme A reductase, has been demonstrated in numerous studies to markedly improve survival rates following radiation exposure in both murine and nonhuman primate models (163–165). In a landmark study (165), male CD2F1 mice administered with GT3 pretreatment exhibited 100% survival rate subsequent to exposure to 11 Gy of cobalt-60 gamma radiation—a dose that induces severe hematopoietic acute radiation syndrome accompanied by moderate gastrointestinal injury. Comprehensive proteomic analysis demonstrated that GT3 significantly modulated radiation-induced alterations in serum protein expression profiles, particularly affecting pathways associated with immune regulation, inflammatory responses, and cellular motility. The unprecedented achievement of complete survival in this animal model establishes GT3 as a prophylactic radioprotective agent, representing a paradigm shift in radiation countermeasure research.

### 4.4 Mesenchymal stem cells (MSCs) and exosomes

Since the first study (198) demonstrating the therapeutic value of MSCs in RIII by Sémont et al. in 2006, related explorations have continued to expand. Multiple studies have shown that MSCs prolong the survival of irradiated mice, with treated animals exhibiting hyperplastic intestinal villi characterized by increased proliferative cells and reduced apoptotic cells in crypts.

MSCs promote the growth of endogenous Lgr5+ ISCs by increasing the activation of the Wnt/β-catenin signaling pathway (199). And they could secrete IL-6 to promote epithelial regeneration (200). The research has found that MSCs increase the levels of serum R-spondin1, KGF, IL-10, and PGE2 to promote cell proliferation and inhibit apoptosis and inflammation (201). Moreover, MSC infusion can accelerate the formation of new blood vessels at radiation-damaged sites through the intrinsic repair effect triggered by the upregulation of expressed SDF-1, VEGF, basic fibroblast growth factor (bFGF), and Flk-1 (202). It is worth noting that MSC-conditioned medium also shows the potential to promote the regeneration of irradiated epithelial tissues (203). Additionally, Lindar et al. reported that repeated autologous transplantation of MSCs in a pig model of radiation-induced proctitis could counteract the inflammation of rectal mucosa by increasing the host’s IL-10 production and reduce collagen deposition by decreasing the expression of local Col1a2/Col3a1 and TGF-β/CTGF and improving the MMP/TIMP balance, protecting the rectum from radiation-induced fibrosis (204). These preclinical studies have demonstrated that MSC-based therapies have strong potential for repairing lesions associated with radiation-induced enteritis.

However, challenges such as tumor immune evasion risk due to MSC-mediated immunosuppression and allogeneic MSC intolerance have spurred interest in extracellular vesicles (EVs) as a safer alternative. Exosome is a type of EVs. Recent research (205, 206) confirms that mesenchymal stem cell-derived exosomes (MSC-exos) promote epithelial repair, maintain intestinal structural integrity, and mitigate radiation-induced gastrointestinal toxicity in mice, offering a promising cell-free therapeutic strategy. Leilei Yang et al. (207) discovered mesenchymal stem cell-derived exosomes (MSC-exos) injection alleviated intestinal inflammation, enhanced epithelial integrity, and upregulated stem cell markers (LGR5, OCT4) in a RIII mouse model. And in irradiated Lgr5^+^ intestinal epithelial stem cells (IESCs), MSC-exos promoted proliferation and suppressed apoptosis. MSC-exos reversed radiation-induced elevation of pro-inflammatory cytokines (TNF-α, IL-6) and miR-195 overexpression. Mechanistically, miR-195 inhibition by MSC-exos activated Akt and Wnt/β-catenin pathways, which are critical for IESC survival and regeneration. Nevertheless, the current body of research in this domain remains limited, and the therapeutic potential of exosomes warrants more extensive investigation through rigorous scientific studies.

### 4.5 Gut microbiome regulation

Probiotics, FMT, and microbial metabolites (e.g., SCFAs) restore intestinal health (95, 97). Probiotics like *Saccharomyces boulardii*, *bifidobacteria*, or *synbiotics* reduce radiocolitis incidence in mice and nonhuman primates. “Elite survivor” mice exposed to lethal radiation exhibit unique microbiota; transferring their gut microbiota to germ-free mice confers radiation resistance. SCFAs (acetate, propionate, butyrate) enhance radiation tolerance via DNA damage reduction and ROS suppression. Metabolomic studies identify tryptophan derivatives (1H-indole-3-carbaldehyde, kynurenine) as long-term Radioprotectors.

Prebiotics and probiotics exhibit synergistic effects in mitigating RIII. Inulin hydrogels (IGs) were employed as carriers for multi-strain probiotics (MSPs) comprising *Clostridium butyricum*, *Bifidobacterium adolescentis*, and *Akkermansia muciniphila* (9). Experimental findings demonstrated that the IG/MSP-based synbiotic formulation exhibited superior protective efficacy against ionizing RIII in comparison to individual probiotic strains and IGs alone. This synbiotic intervention effectively restored multiple physiological parameters to near-normal levels, including locomotor activity, intestinal barrier integrity (as evidenced by occludin and ZO-1 expression), histological damage, pro-inflammatory cytokine concentrations, gut microbiota composition, and short-chain fatty acid production. These collective data indicate that IGs significantly potentiate the therapeutic effects of probiotics in mitigating RIII.

Genetically Engineered Probiotics: With the progressive development of synthetic biology and the interdisciplinary integration of medical sciences, genetically engineered bacteria have exhibited remarkable potential in radiation-induced intestinal injury (RIII) precision therapy through the customization of microbial functionalities. D. F. Hamade et al. (10) engineered the second-generation probiotic *Lactobacillus reuteri* (LR-IL-22), which facilitates targeted delivery of interleukin-22 (IL-22) to mitigate intestinal damage, demonstrating significant enhancement of intestinal barrier integrity post-radiotherapy (P = 0.0167). This intervention effectively reduced concentrations of radiation-induced pro-inflammatory cytokines, specifically KC/CXCL1 (P = 0.002), IFN-γ (P = 0.0024), and plasma Eotaxin/CCL11 (P = 0.0088), while maintaining the population of Lgr5+GFP + intestinal stem cells (P = 0.0010) to facilitate intestinal regeneration. Moreover, in murine models with disseminated 2F8cis ovarian cancer, the combination of LR-IL-22 with fractionated radiotherapy (6.5 Gy × 4 fractions) resulted in substantial tumor burden reduction. When administered in conjunction with paclitaxel/carboplatin chemotherapy, this approach further improved survival outcomes. In addition to LR-IL-22, their research further validated that LR-IFN-β serves as an efficacious therapeutic agent for intestinal radioprotection and radiation damage mitigation (11). Their short half-life balances efficacy and safety, addressing bioagent retention challenges.

Overall, the engineered bacteria demonstrate dual therapeutic efficacy, functioning both as Radioprotectors to prevent radiation-induced damage and as mitigators to alleviate existing injuries, thereby transcending conventional single-mode protective strategies. This innovation establishes a novel “probiotics-cytokine synergistic radio-sensitization” paradigm that integrates microbial therapy with cytokine modulation to enhance radiation efficacy. Additionally, regarding the research on probiotics and FMT in clinical trials, it will be discussed in the “Translational Research Highlight” section.

## 5 Translational Research Highlight

Since increased attention has been paid to gastrointestinal symptoms associated with radiotherapy, a growing number of researchers have initiated clinical trials in an effort to facilitate clinical translation. We have compiled all currently accessible relevant clinical trials into Table 3.

**TABLE 3:** Summary of the clinical trials.

Study Start	Study title	Trial ID	Status^a^	Sponsor	Condition	Study design	Inclusion criteria	Intervention	Primary outcome measures
2025.06	Gegen Qinlian Tang and Probiotics for Radiation Enteritis	NCT06836960	Enrolling by invitation	Jiujiang No.1 People’s Hospital	Acute Radiation Enteritis	Interventional; Phase I/II	1.Age:Participants aged 18–80 years 2.Diagnosis:Patients diagnosed with malignant tumors confirmed by cytological or pathological examination.Patients scheduled for abdominal or pelvic radiotherapy based on treatment guidelines. 3.Cognitive and Communication Ability:Participants must have clear consciousness and normal cognitive abilities to communicate effectively. 4.Consent:Participants must provide written informed consent and agree to participate in the study	Drug: Probiotic Therapy with Gegen Qinlian Tang	Incidence of Acute Radiation Enteritis
2024.05	Umbilical-Cord-Derived Mesenchymal Stem Cells Injection for Chronic Radiation Proctitis	NCT06438809	Recruiting	Jiangsu Topcel-KH Pharmaceutical Co., Ltd	Chronic Radiation Proctitis	Interventional; Phase I/II	1. Fully understand and sign the informed consent form; 2. Age ≥18 years and <80 years; 3. Good physical condition (WHO performance status score 0–1); 4. Pathologically diagnosed with pelvic malignant tumors and received radiotherapy; 5. Diagnosed with chronic radiation proctitis after at least 6 months of endoscopic examination following the completion of radiotherapy and ineffective conventional treatment; 6.Screening period LENT-SOMA score ≥1; 7.Subjects and their spouses or partners have no plans for conception from screening to 6 months after the end of the trial, no plans for sperm or egg donation, and agree to use effective non-pharmacological contraceptive measures during the trial	Biological: Mesenchymal Stem Cells (MSCs)	1.The severity of adverse events after administration as assessed by CTCAE v5.0. 2.The incidence of adverse events after administration
2024.04	Efficacy of Hyaluronic Acid in Prevention of Acute Radiation Proctitis	NCT06469216	Completed	Assiut University	Acute Radiation Proctitis; Acute Radiation Enteritis	Interventional; Phase I/II	1.All patients diagnosed with pelvic or gastrointestinal malignancies (tumors in uterus, cervix, prostate, seminal vesicles, kidney, colon, etc.); 2. All patients who required adjuvant or radical radiation therapy; 3. Age˂ 80 years; 4. Karnofsky Performance Status ≥60	Drug: Hyaluronic Acid; Other: Placebo	Time (in days) to occurrence of Acute Radiation Proctitis
2022.01	Randomized clinical study on evaluation of Kangfuxin liquid in the prevention and treatment of cervical cancer radiation enteritis	ChiCTR2200055355	Completed	Shanghai Sixth People’s Hospital	Cervical cancer radiotherapy-induced proctitis	Interventional; Phase: III/IV	1. Patients with cervical cancer first diagnosed by pathology; 2. Patients with stage IB-IV (2019 FIGO stage) requiring radiotherapy; 3. Aged 18–70 years; 4. Have not received pelvic radiotherapy before enrollment; 5. ECOG general condition score 0–2; 6. The expected survival period is more than 12 months; 7. Laboratory tests before radiotherapy and chemotherapy meet the following conditions: (1) Liver function: total bilirubin ≤1.5 times the upper limit of normal (ULN); AST and ALT ≤2.5 times the upper limit of normal (ULN); alkaline phosphatase≤1.5 times the upper limit of normal (ULN); (2) Renal function: creatinine clearance rate ≥80 mL/min; (3) Blood function: absolute neutrophil count (ANC) ≥ 2 × 10^9/L, platelet count ≥100 × 10^9/L and hemoglobin ≥ 9 g/dL; 8. Agree and sign the informed consent form (if the patient himself has no judgment ability, his legal guardian is required to sign the informed consent form)	Drug: Kangfuxin liquid	Number of stools
2009.01	Prevention of Acute Radiation Enteritis With Glutamine	NCT00828399	Completed	Castilla-León Health Service	Acute Radiation Enteritis	Interventional; Phase IV	1.Age >18 years; 2. Gynaecological, prostatic, rectal or other abdominal cancer; 3. Radiotherapy with/without chemotherapy	Dietary Supplement: Glutamine (Oral glutamine 30g/day); Dietary Supplement: Whole protein (Oral whole protein 30 g/day)	Number of patients with acute radiation enteritis
2008.01	Fecal Microbiota Transplantation for Radiation Enteritis	NCT03516461	Recruiting	The Second Hospital of Nanjing Medical University	Radiation Enteritis	Interventional; Phase:Not Applicable	1.Age ≥18 years old; 2. Radiation enteritis diagnosed by colonoscopy after finishing radiotherapy	Procedure: Fecal Microbiota Transplantation (FMT)	Clinical response rate
2006.12	Impact of Probiotics BIFILACT^®^ on Diarrhea in Patients Treated with Pelvic Radiation	NCT01839721	Completed	CHU de Quebec-Universite Laval	1.Cancer 2.Diarrhea 3. Abdominal Pain	Interventional; Phase I/II	1.They had a pelvic cancer: gynecologic, rectal, or prostatic. 2. They were to receive radiotherapy treatments for a minimum of 40 Gy at the pelvic level, with or without chemotherapy. 3. They had Eastern Cooperative Oncology Group(ECOG) performance status of 0 or 1	Drug: Bifilact^®^; Other: placebo	testing the efficacy of probiotics Bifilact^®^, in comparison to a placebo to assess its ability to prevent or delay the incidence of moderate or severe symptoms of diarrhea during the period of treatment by radiotherapy
2003.12	Octreotide in Preventing or Reducing Diarrhea in Patients Receiving Chemoradiotherapy for Anal or Rectal Cancer	NCT00075868	Completed	Radiation Therapy Oncology Group	Anal Cancer; Colorectal Cancer; Drug/Agent Toxicity by Tissue/Organ	Interventional; Phase III	DISEASE CHARACTERISTICS: Histologically confirmed primary anal or rectal cancer No metastasis beyond the pelvic regional nodes Must be scheduled to receive chemoradiotherapy PATIENT CHARACTERISTICS: Age:18 and over	Drug: octreotide acetate; Other: Placebo	Prevention of the incidence of moderate, severe, or life-threatening diarrhea
1999.06	Pentosan Polysulfate in Treating Patients with Gastrointestinal Disturbance Caused by Radiation Therapy	NCT00003825	Completed	Radiation Therapy Oncology Group	Diarrhea; Radiation Enteritis	Interventional; Phase I/II	DISEASE CHARACTERISTICS: Must have received prior radiotherapy to the abdomen and pelvis and now have radiation related gastrointestinal symptoms (which were not present before the radiotherapy and/or attributed to other causes) Proctitis Diarrhea Melena (blood in stools) Severity of symptoms classified as grade 1, 2, or 3 PATIENT CHARACTERISTICS: Age: Any age Performance	Drug: pentosan polysulfate sodium; Procedure: quality-of-life assessment	—

^a^ The “Status” refers to the status as of 24 July 2025.

### 5.1 Traditional Chinese medicine (TCM)

The aforementioned therapeutic approach has exhibited substantial therapeutic potential in both cellular and animal models. To date, preliminary clinical translation efforts have yielded promising results, paving the way for further advancement.

In clinical practice, the herbal retention enema targeting RIII has achieved certain therapeutic effects. The base ingredients of the modified Baitouweng decoction (mBTWD) are mainly Baitouweng decoction, which consists of four herbs: Pulsatilla (Baitouweng), Coptis chinensis (Huanglian), tractat (Huangbo), and ash bark (Qinpi). Zihong Wu et al. (12) conducted a search for relevant randomized controlled trials (RCTs) in PubMed, Embase, Cochrane Library, Web of Science, CNKI, Wanfang Database, SionMed and China Science Journal Database. Eventually, 17 studies were included, involving a total of 1611 patients. mBTWD demonstrates significant efficacy in improving patients’ TCM syndrome scores ( MD=−3.41,P<0.00001 ), alleviating intestinal symptoms ( RR=1.23,P=0.0001;OR=3.51,P<0.00001 ), and optimizing biochemical parameters (C-reactive protein, Karnofsky performance scale, occult fecal blood) ( P<0.05 ). Network pharmacology and molecular docking analyses have identified that the core bioactive components of mBTWD, including quercetin, isorhamnetin, and β-sitosterol, potentially exert therapeutic effects through targeting key molecular pathways involving MYC, TP53, and MAPK14/MAPK1.

A Phase I/II clinical trial (NCT06836960) titled “Gegen Qinlian Tang and Probiotics for Radiation Enteritis” is currently being conducted at Jiujiang No.1 People’s Hospital (China). This trial aims to recruit 60 patients with malignant tumors undergoing abdominal or pelvic radiotherapy. Participants will be randomly allocated into one of three groups: the control group (no intervention), the probiotics-only group (Bifidobacterium Tricell Capsules, administered twice daily at three capsules per dose), and the combination therapy group (probiotics combined with symptom-adapted Gegen Qinlian Tang). The primary outcome measure will focus on the incidence and severity of acute radiation enteritis, assessed using the RTOG/EORTC grading criteria (Grades 0–IV). Observations will be recorded daily during the radiotherapy period, with a follow-up duration of 3 months post-treatment. This study aims to provide evidence supporting the use of probiotics and herbal medicine as effective strategies to mitigate radiotherapy-induced side effects and enhance patient outcomes.

### 5.2 Other compounds and biomolecules

A randomized, double-blind, non-inferiority clinical trial (13) enrolled patients presenting with grade 2 or 3 diarrhea (as per the Common Terminology Criteria for Adverse Events version 4.0) following radical radiotherapy for pelvic malignancies. The study aimed to compare the therapeutic efficacy and safety profiles of the antisecretory agent Racecadotril versus the antispasmodic medication Loperamide in managing acute radiation enteritis. Between 2019 and 2022, a total of 162 patients were randomized into treatment groups. In the intention-to-treat analysis, 81 patients in the Racecadotril cohort demonstrated improvement from grade 2 or 3 diarrhea to grade 1 or 0 diarrhea (68 patients, 84%), while 70 patients (86.4%,) in the Loperamide group exhibited comparable clinical improvement (*P* = 0.66 > 0.05). The intergroup difference in improvement proportions was 2.4% (95% confidence interval [CI]: −8.5%–13.4%). As the upper limit of the 95% CI exceeded the predefined non-inferiority margin of 10% (13.4%), the non-inferiority of Racecadotril relative to Loperamide could not be established. Nevertheless, Racecadotril emerges as a preferred therapeutic option for acute radiation enteritis due to its preservation of intestinal motility, comparable clinical efficacy to Loperamide, and a more favorable adverse effect profile.

From June 2021 to January 2023, 86 patients with cervical cancer who received radiotherapy were included in a clinical trial (ChiCTR2200055355) (14) on the efficacy of kangfuxin liquid retention enema in alleviating RIII in cervical cancer patients. These patients were divided into two groups according to the treatment method: the kangfuxin liquid retention enema observation group and the control group, with 43 cases in each group. The results showed that the incidence of RIII in the observation group after radiotherapy was significantly lower, at 20.93%, while that in the control group was 41.86%. The average onset time of RIII in the observation group was significantly longer than that in the control group (*P* < 0.05). The incidence of moderate to severe radiation-induced enteritis in the observation group was significantly lower, at 2.33%, while that in the control group was 18.60%. In addition, after radiotherapy, the levels of CD3 and CD4 in the observation group increased, while the level of CD8 decreased, showing statistical differences compared with the control group (*P* < 0.05). The levels of neutrophils and C-reactive protein (CRP) in the observation group were lower than those in the control group, and the differences were statistically significant (*P* < 0.05). Thus, it can be concluded that the rehabilitative fluid retention enema therapy shows efficacy in delaying the onset of RIII and reducing its prevalence, thereby improving the clinical manifestations of cervical cancer patients after radiotherapy. This therapeutic effect can be attributed to the enhancement of immune function and the reduction of inflammatory mediators.

Similarly, a recent randomized controlled clinical trial evaluating the efficacy of hyaluronic acid enema in preventing acute radiation proctitis in cancer patients (especially those diagnosed with abdominal and pelvic tumors and subsequently requiring radiotherapy) has been completed (NCT06469216). However, no relevant data has been published so far. However, we are willing to believe that hyaluronic acid enema is as effective as kangfuxin liquid in treating acute radiation proctitis. A meta-analysis indicates that for patients with symptoms of vaginal and anorectal mucositis, local application of hyaluronic acid after radiotherapy and chemotherapy is an innovative approach for clinical treatment, which helps patients cope with adverse reactions (15).

A clinical trial (162) involving 10 patients with RIII treated with a combination of pentoxifylline and vitamin E found that the combined treatment could reduce symptoms by 41% and 80% respectively at 6 months and 18 months. *In vitro*, pentoxifylline and ascorbyl phenol synergistically inhibited the expression of TGF-β1 protein and mRNA. This inhibitory effect was achieved at the transcriptional level and led to subsequent inhibition of TGF-β1/Smad targets (Col Iα1, FN1, PAI-1, CTGF).

Notably, a substantial number of clinical trials investigating RIII failed to demonstrate anticipated outcomes. Alfonso Vidal-Casariego et al. (16) conducted a randomized, double-blind, Phase III clinical trial (NCT00828399) to evaluate the efficacy of glutamine in preventing acute radiation enteritis, which ultimately yielded negative findings. Similarly, Phase III randomized controlled trials investigating octreotide for diarrhea prevention during radiotherapy and chemotherapy for rectal cancer (NCT00075868) (208) and pentosanpolysulfate for managing radiation-induced diarrhea and hemorrhage (NCT00003825) (209) both produced unfavorable results.

### 5.3 Mesenchymal stem cells (MSCs)

MSC therapy has been applied to patients with severe gastrointestinal complications following radiotherapy (210). Surprisingly, in a case series by Voswinkel et al. (211), four patients achieved cessation of rectal bleeding, fistula healing, pain relief, and no tumor recurrence after MSC treatment.

A multicenter Phase I/II trial (NCT06438809) is currently evaluating this approach for chronic radiation-induced injuries refractory to conventional therapies. Zhaoshen Li et al. plan to enroll patients who fulfilled the inclusion criteria, had received radiotherapy followed by unsuccessful conventional treatment, and were diagnosed with chronic radiation proctitis through endoscopic examination conducted at least 6 months post-treatment. These patients will be administered umbilical cord-derived mesenchymal stem cells (120 million cells) via injection. The therapeutic response is monitored over a 24-month period following the administration. The primary endpoints include: the severity of adverse events assessed using CTCAE v5.0 within 28 days post-injection and the incidence of adverse events, with particular emphasis on evaluating the safety profile of MSC treatment for radiation proctitis. Additionally, on day 112 post-treatment, the improvement and recurrence rates of chronic radiation proctitis are evaluated to determine the efficacy of MSC therapy. This research outcome has the potential to substantially accelerate the clinical translation and application of MSC.

### 5.4 Gut microbiome regulation

A probiotic containing live *lactobacillus* acidophilus plus bifidobacterium bifidum was administered as an investigational agent in a double-blind clinical trial targeting RIII in patients diagnosed with locally advanced cervical carcinoma. In the placebo cohort (*n* = 31), 45% of participants manifested grade 2–3 diarrhea, whereas in the experimental group (*n* = 32), this incidence was reduced to 9% (*P* = 0.002). The utilization of anti-diarrheal medications in the placebo arm demonstrated a significant reduction(*P* = 0.003).

Furthermore, stool consistency in the treatment group exhibited marked improvement (*P* < 0.001). These findings substantiate that the probiotic containing live *lactobacillus* acidophilus plus bifidobacterium bifidum effectively mitigates the occurrence of diarrhea (212).

And another randomized, double-blind, placebo-controlled clinical trial (NCT01839721) was conducted to assess the efficacy of the probiotic formulation Bifilact^®^ in managing moderate to severe therapeutic diarrhea during pelvic radiotherapy. A cohort of 246 patients was randomly allocated to receive either placebo or one of two dual-strain Bifilact^®^ probiotic formulations (containing *Lactobacillus* LAC-361 and Bifidobacterium BB-536) at a standard dosage of twice daily (130 billion CFU). The findings indicated that the standard dosage of Bifilact^®^ potentially reduces the incidence of grade 2-3-4 radiation-induced diarrhea by the conclusion of treatment in patients undergoing pelvic radiotherapy (213).

It is particularly noteworthy that Xiao Ding et al. conducted a pioneering clinical investigation (NCT03516461) into FMT as a therapeutic intervention for chronic radiation enteritis (CRE) (214). Between January and November 2018, five female patients (median age: 58 years; range: 45–81 years) with a median baseline RTOG/EORTC grade of 2 (range: 2–4) underwent FMT treatment. The therapeutic outcomes revealed that three patients exhibited significant clinical improvement, manifesting as reduced diarrhea, diminished rectal bleeding, alleviated abdominal/rectal pain, and improved fecal incontinence, accompanied by enhanced Karnofsky Performance Status (KPS) scores. Importantly, the procedure demonstrated an excellent safety profile, with no mortality or infectious complications associated with FMT observed throughout the study. During the extended follow-up period (8–18 months), only one mild FMT-related adverse event was documented. Furthermore, 16S rRNA sequencing analysis provided compelling evidence that FMT effectively modulated the intestinal microbiota composition in the treated patients. Currently, this clinical trial remains in the active recruitment phase (RECRUITING), and we anticipate the involvement of additional patients to strengthen the robustness of the research evidence.

## 6 Future perspectives

**FIGURE 3:**
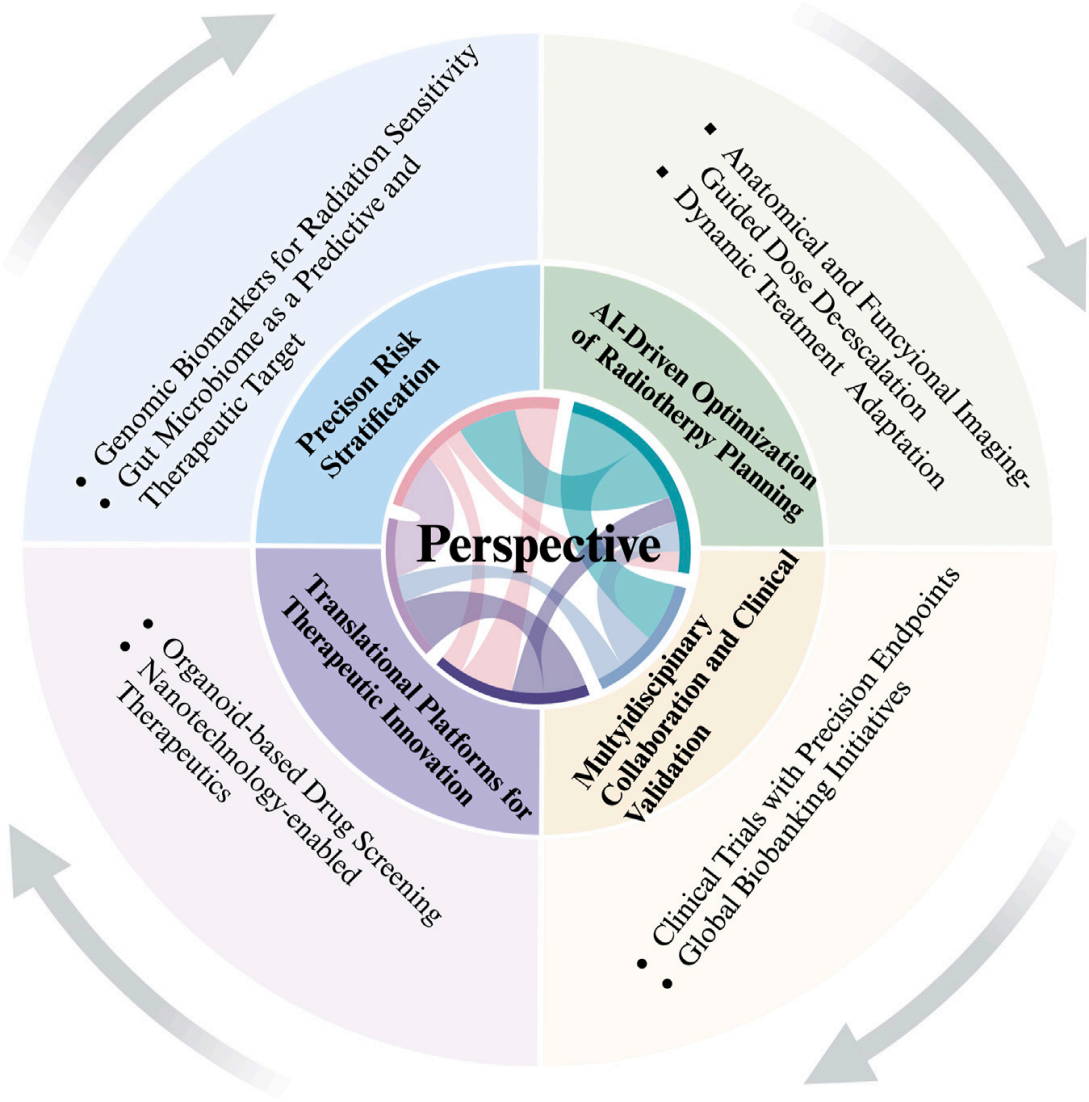
Future Perspectives The figure was created in https://BioRender.com.

To address the unmet clinical needs of RIII, a precision medicine framework integrating​genomics-driven risk stratification, microbiome-based diagnostics, and AI-optimized radiotherapy planning represents a transformative paradigm. Below outlines actionable strategies for achieving this vision (Figure 3):

### 6.1 Precision risk stratification via genomics and microbiome profiling

#### 6.1.1 Genomic biomarkers for radiation sensitivity

Multi-gene panels for personalized risk assessment: Develop diagnostic panels incorporating radiation-responsive genes (e.g., ATM, TP53, XRCC1/2/3, NRF2) and single-nucleotide polymorphisms (SNPs) linked to DNA repair efficiency (e.g., XRCC1 399 Arg/Gln) and apoptosis regulation (e.g., P53 Pro/Arg72). These panels could predict individual susceptibility to acute mucositis, chronic fibrosis, or secondary malignancies.

Epigenetic biomarkers: Leverage DNA methylation signatures (e.g., NRF2-regulated genes) and histone modification patterns (e.g., H3K27ac in stem cell niches) to identify epigenetic dysregulation predisposing patients to RIII. Liquid biopsy-based methylation assays could enable non-invasive monitoring.

Pharmacogenomics for Radioprotectors: Identify genetic determinants of response to mitigators like PUMA inhibitors, HDAC inhibitors (e.g., TSA), or NRF2 activators (e.g., bardoxolone), optimizing drug selection for high-risk patients.

#### 6.1.2 Gut microbiome as a predictive and therapeutic target

Microbiome-based risk prediction: Establish fecal microbiome signatures (e.g., decreased *Lactobacillus/Akkermansia* and increased Enterobacteriaceae) as early biomarkers for radiation enteropathy severity. Machine learning models integrating microbiome composition, metabolomics (e.g., SCFA levels), and host gene expression could improve prognostic accuracy.

Microbiome modulation for prevention: Engineer synbiotics or FMT protocols to enrich beneficial taxa (e.g., *Clostridium* XIVa clusters) and suppress pro-inflammatory/pathogenic species. Preclinical evidence suggests modulation of the gut microbiome can attenuate radiation-induced dysbiosis and fibrosis.

### 6.2 AI-driven optimization of radiotherapy planning

#### 6.2.1 Anatomical and functional imaging-guided dose de-escalation

Organ-at-risk (OAR) segmentation: Utilize deep learning algorithms (e.g., convolutional neural networks) to automate segmentation of the small intestine and adjacent organs in CT/MRI scans, improving dose-sparing precision in intensity-modulated radiotherapy (IMRT) or proton therapy.
Predictive modeling of radiation toxicity: Train machine learning models on multi-parametric data (dose distribution, tissue oxygenation, microbiome profiles) to predict RIII incidence and severity. For example, a model integrating dose-volume histograms (DVHs) with gut microbiome alpha-diversity could identify patients at high risk for chronic fibrosis, prompting dose adjustments or adjuvant therapies.

#### 6.2.2 Dynamic treatment adaptation

Real-time adaptive radiotherapy: Deploy AI-driven platforms to monitor anatomical changes (e.g., intestinal edema, fibrosis progression) during radiotherapy via weekly MRI or cone-beam CT. Adjust radiation beams dynamically to avoid high-dose exposure to injured regions.
Synthetic lethality strategies: Use AI to identify synthetic lethal interactions between radiation and specific gene mutations (e.g., ATM-deficient tumors) or microbiome profiles, enabling targeted combination therapies (e.g., PARP inhibitors for ATM-mutant cancers with concurrent gut radioprotection).

### 6.3 Translational platforms for therapeutic innovation

Organoid-based drug screening: Develop patient-derived intestinal organoids co-cultured with personalized gut microbiota to test radioprotective compounds (e.g., NRF2 agonists, exosome-based therapies) and identify responders/non-responders.
Nanotechnology-enabled therapeutics: Engineer nanoparticles targeting radiation-damaged crypts (e.g., Lgr5+ stem cells) to deliver NRF2 activators, DNA repair enzymes, or anti-inflammatory cytokines (e.g., IL-10) selectively to injured tissues.

### 6.4 Multidisciplinary collaboration and clinical validation

Clinical trials with precision endpoints: Design randomized trials stratifying patients by genomics/microbiome risk scores to evaluate the efficacy of tailored interventions (e.g., FMT plus IL-22 analogs vs standard care).
Global biobanking initiatives: Establish international consortia to collect longitudinal multi-omics data (genomics, transcriptomics, metabolomics, microbiome) from irradiated patients, enabling discovery of novel biomarkers and therapeutic targets.
